# Therapeutic impact of human trophoblast stem cells in peritoneal and pneumonia-induced sepsis in mice

**DOI:** 10.1186/s13287-025-04479-z

**Published:** 2025-07-21

**Authors:** Narae Hwang, Gu Li, Ekaterina Murzin, Cassidy J. Mays, James A. Lederer, Xiaoli Liu, Mark A. Perrella

**Affiliations:** 1 Division of Pulmonary and Critical Care Medicine, Department of Medicine, Brigham and Women’s Hospital, Harvard Medical School, 75 Francis Street, Boston, MA USA; 2 Division of Pediatric Newborn Medicine, Department of Pediatrics, Brigham and Women’s Hospital, Harvard Medical School, Boston, MA USA; 3 Department of Surgery, Brigham and Women’s Hospital, Harvard Medical School, Boston, MA USA

**Keywords:** Trophoblast stem cells, Sepsis, Acute organ injury, Inflammation, Bacterial clearance

## Abstract

**Background:**

Sepsis is a complex and life-threatening disease process related to a systemic response to severe infection. Due to the challenges of treating patients with sepsis, new therapies are being investigated, including cell-based approaches. Trophoblast stem cells (TSCs) are immune privileged cells with immunomodulatory properties. Thus, we proposed that TSCs may be beneficial in experimental models of sepsis to regulate the immune response and curtail organ injury.

**Methods:**

Sepsis was induced by experimental models in mice; cecal ligation and puncture (CLP) and lung infection with *Streptococcus (S.) pneumoniae.* TSCs were isolated from the chorionic villi of human (h) term placentas, and from mouse (m) placentas using anti-CD117 MicroBeads, and were administered intravenously 6 h after CLP or *S. pneumoniae* infection. We assessed mortality, bacterial clearance, organ injury, inflammatory response, and production of cytokines and chemokines.

**Results:**

CD117^+^ hTSCs did not express human leukocyte antigen (HLA) I or II, and were clonogenic and self-renewing. CLP led to severe mortality by 7 days, and administration of either hTSCs or mTSCs resulted in markedly improved survival compared with control cells or vehicle. hTSCs promoted bacterial clearance and decreased organ injury in the liver, kidney, spleen, and bowel. The elevated innate immune response in the peritoneum, predominantly neutrophils, was attenuated by hTSCs. In addition, neutrophil infiltration into the spleen was less in mice receiving hTSCs, which corresponded with reduced plasma pro-inflammatory cytokines and chemokines. When assessing the lung response to *S. pneumoniae* infection, administration of hTSCs resulted in fewer bacteria in bronchoalveolar lavage fluid (BALF) and lung tissue, and less lung edema and injury. Neutrophils, which were markedly increased in BALF, were diminished and infiltration of neutrophils and macrophages into the lungs was decreased by hTSCs. BALF pro-inflammatory cytokines and chemokines were mitigated by hTSCs to levels of Sham mice, and systemic injury to the liver and spleen was attenuated.

**Conclusions:**

CD117^+^ hTSCs are immune privileged cells that when given after the onset of experimental models of infection/sepsis resulted in improved outcomes due to enhanced bacterial clearance, resolving inflammation, and less organ injury. These data support hTSCs as a potential cell-based therapy for sepsis.

**Supplementary Information:**

The online version contains supplementary material available at 10.1186/s13287-025-04479-z.

## Background

Sepsis is a complex disease process due to the systemic response to infection, and the leading cause of morbidity and mortality in intensive care units [1–5]. Due to the heterogeneous nature of sepsis, and the therapeutic challenges with management being predominantly antibiotics and supportive care, there is a need to develop new strategies to advance the treatment of sepsis and its related complications. New advances are presently being explored, including cell-based therapies [6].

The challenge in the treatment of sepsis is that it is a very heterogeneous clinical process, resulting in multiorgan injuries, thus targeting a specific biological pathway for everyone is challenging [7]. Rapid and early phenotyping of patients by clinical and immune endotypes, is being investigated to address these therapeutic challenges with a personalized approach [8]. We are proposing that giving a cell-based therapy will allow viable therapeutic cells to sense the underlying septic environment, and respond accordingly with varied actions. A focus of early phase clinical trials has included the use of mesenchymal stem/stromal cells (MSCs) for sepsis [9, 10], and also for the complication of acute respiratory distress syndrome (ARDS) [11]. While our laboratory and others have demonstrated that MSCs improve outcomes in preclinical models of bacterial sepsis [12–20], we seek to further explore additional candidate stem cells to control sepsis and its accompanying pathobiology. This includes cells with easy access for harvest, and also immune privilege, thus allowing the use of allogeneic cells for treatment.

Recently our laboratory identified a CD117^+^ trophoblast stem cell (TSC) derived from the fetal portion of near-term mouse (m) placentas [21]. These cells were clonogenic, self-renewing, and multipotent with the capability of differentiating into cells of the endoderm, mesoderm, and ectoderm. The mTSCs also showed an absence of major histocompatibility complexes I and II, demonstrating immune privilege. Moreover, when these cells were administered to mice after the onset of acute lung injury due to bleomycin exposure, they resulted in a decrease in inflammation in the lungs and a reduction of parenchymal cell death [22]. Thus, we propose to advance our investigation into therapy for sepsis and associated organ injury using TSCs harvested from term human placentas. To assess the impact of human (h) TSCs on the pathobiology of experimental sepsis, we evaluated two different models; cecal ligation and puncture (CLP) model of polymicrobial peritoneal sepsis [23], and a pneumonia model due to *Streptococcus (S.) pneumoniae* and subsequent sepsis [24].

## Material & method

### Isolation of hTSCs

A human term placenta was naturally delivered and clinically confirmed to be normal. The portion of the placenta containing chorionic villi on the fetal side was harvested. Chorionic villi were obtained by trimming the surrounding tissue. Cytotrophoblast cells were isolated following a 3-step sequentially digested in HBSS buffer containing 0.75% NaHCO3, 25 mM HEPES, 0.25% Tyrosine and ~ 300 U/mL DNase at 37 °C as previously described [25]. The combined resuspensions were centrifuged at 1000 × g for 10 min, and the pellet was suspended in 6 ml CMF-Hank’s solution. The cell suspension was layered onto Percoll gradients and centrifuged at 1200 × g for 20 min at room temperature with Break ~ 0 and Acceleration ~ 0. The fraction of cytotrophoblast cells in the layer between 15 and 24 ml graduations (1.046–1.065 markers) of a 50 mL-Conical centrifuge tube were collected and centrifuged at 1000 × g for 5 min, which yielded ≈ 1.5 ~ 3 × 10^8^ cells per 40 g of tissue. The subpopulation of CD117^+^ cytotrophoblast cells (hTSCs) were isolated using anti-human CD117 MicroBeads (Miltenyi Biotec, #130-091-332) by MACS separation system and were expanded in DMEM/F12 (Lonza, #12–719 F) medium supplied with 10% FBS, 25 nG/mL hFGF (PeproTech, #100-18B) and 1% Penicillin/Streptomycin [21, 22].

### Isolation of mTSCs and mFibroblasts (mFBs)

mTSCs were harvested from placentas that were collected at embryonic day 18.5 from pregnancies of wild-type C57BL/6 mice as described [21]. mFBs were harvested from the lungs of wild-type adult mice, and isolated (lineage negative/Sca1-depleted cells) as described [16].

### Characterization of hTSCs

hTSCs were expanded in culture, and were also plated at 100 cells per 100 mm diameter dish (low density, ≈ 1 cell per 60 mm^2^) to obtain multicellular clones derived from a single founder cell. The clones were stained for CD117 and DAPI (4’,6-diamidino-2-phenylindole) at day 10. For flow cytometry assays, hTSCs were detached from culture dishes using HyQtase and blocked with Fc (hCD32/16) at room temperature for 15 min, followed by incubating with primary antibodies conjugated with fluorescence at 4 °C for 30 min in darkness [21]. The cells were then assessed using BD FACS Canto II, and the data were analyzed using FlowJo software (TreeStar). Analyses included hCD73 (BioLegend, Cat.344015), hCD90 (BioLegend, Cat.555596), hCD105 (BioLegend, Cat.323207), hCD11b (BioLegend, Cat.301310), hCD31 (BioLegend, Cat.303109), hCD34 (BioLegend, Cat.343611), hCD45 (BioLegend, Cat.368516), human leukocyte antigen (HLA) I (BioLegend, Cat. 311409), and HLA II (BioLegend, Cat.361715).

### Mouse models of sepsis and cell administration

#### Cecal ligation and puncture (CLP)

C57BL/6 male mice (Charles River), 7–9 weeks of age, underwent Sham and CLP surgery as described [16, 26]. In brief, a ligation was placed at two-thirds of the cecum, and two holes were punctured with a 21-gauge needle.

#### Pneumonia

Clinically isolated *S. pneumoniae* (strain 99.55 capsular subtype 6 A-intranasal route [27]) were suspended in PBS, at a final concentration of 6.25 × 10^8^ colony-forming units (CFUs) in 1 mL PBS. C57BL/6 male mice (Charles River), 7–9 weeks of age, were intranasally inoculated with 40 µL bacteria suspension (2.5 × 10^7^ bacteria) per mouse.

#### Administration of cells

The mTSCs and mFBs (for survival assessment in CLP model) and hTSCs (for both models of CLP and *S. pneumoniae* lung infection) were suspended in PBS containing 100 units/mL heparin, at a final concentration of 2.5 × 10^6^/mL. Mice were intravenously injected via tail vein with 200 µL of cell suspension (5 × 10^5^ for hTSCs, mTSCs, and mFBs) or 200 µL vehicle (PBS + heparin) at 6 hours after CLP or *S. pneumoniae* lung infection. Mice were monitored over a 7 day period after injection of cells to determined survival for CLP-induced sepsis. The severity of illness in the CLP model (two-thirds of the cecum ligated and punctured with two 21-gauge holes) occurs rapidly, with death beginning within 24 h. In the pneumonia model of pneumosepsis using *S. pneumoniae*, the onset of illness is more gradual [27]. Thus, the mice were sacrificed at 24 h after CLP and at 72 h after *S. pneumoniae* infection for additional assessments of immune response and organ injury.

### Harvest of peritoneal fluid (PF)

Mice were anesthetized and the skin of the lower right abdomen was opened. Ten mL of PBS was injected into the peritoneal cavity. After shaking the mouse body to wash the peritoneal cavity well, an ≈ 8 mL PF was recovered. The total cells in PF were counted. PF was also used for flow cytometry to assess the differential of immune cells, as previously described [20].

### Harvest of mouse Bronchoalveolar lavage fluid (BALF) and lung tissue

Anesthetized mice were thoracotomized and the right bronchus was ligated off. Four lobes of the right lung were removed: the superior lobe for bacterial colony counts, the middle lobe for wet-dry weight ratio, and the inferior and posterior-caval lobes were quickly frozen in liquid nitrogen. For BALF collection, 200 µL PBS was injected into the left lung through the trachea and left main stem bronchus and a lavage performed. This procedure repeated 3 times, with a total of ≈ 600 µL BALF collected. The total cells in BALF were counted. BALF was used for flow cytometry to assess differential of immune cells, and for Luminex MAGPIX assay system to profile cytokines and chemokines, as previously described [28]. For lung histological assessment, the left lung was injected with 10% formalin and the trachea was ligated to keep formalin in the lung. The left lung was additionally fixed in 10% formalin for 24 h.

### Bacterial CFUs and inflammatory cell analyses

PF and blood were drawn 24 h after CLP. BALF and the superior lobe of the right lung were harvested 72 h after *S. pneumoniae* infection. The superior lobe was harvested into 1 mL of PBS and homogenized using a tissue homogenizer (Tissue-Tearor, BioSpec Products). Serial dilutions of whole blood, PF, BALF and lung tissue were performed and then incubated overnight at 37 °C on LB agar plates. CFUs of bacteria were counted and calculated as described [16]. Cells from the remaining PF and BALF were stained with antibodies targeting Ly6G-FITC and F4/80-APC, to identify neutrophils and macrophages respectively. The cells were then assessed by flow cytometry using a BD FACS Canto II, and analyzed by FlowJo software.

### Neutrophil isolation and phagocytosis assay

To isolate mouse neutrophils, mice were given an intraperitoneal injection of Bio-Gel P100 polyacrylamide beads (2% solution, Bio-Rad) as described [16]. Twelve to sixteen hours after Bio-Gel administration, neutrophils were isolated and activated with 10 ng/mL G-CSF for 2 h. Following activation, hTSCs were added at a ratio of 1 hTSCs to 5 neutrophils. Green fluorescent protein-labeled *Escherichia (E.) coli* (strain MMB 1287) or FITC-labeled *S. pneumoniae* (strain 99.55) bacteria were then added at 10 multiplicity of infection per neutrophil and incubated in 37 °C for 30 min. Bacterial phagocytosis was measured by flow cytometry as described previously [16, 17, 20].

### Histology and immunohistochemistry

Mice were sacrificed 24–72 h following CLP or *S. pneumoniae* infection, respectively. The organs were harvested and fixed in 10% formalin, processed and embedded in paraffin, and sectioned (5 μm). Lung structure was assessed by hematoxylin and eosin (H&E) stain, or terminal deoxynucleotidyl transferase-mediated dUTP nick end-labeling (TUNEL) staining was performed in the spleen, lung, and bowel, and positive cells in the tissue sections were assessed as described [20–22]. Spleens and lungs were immunostained for Ly6G, and lungs were also stained for CD68, for assessment of neutrophil or monocyte/macrophage infiltration, respectively. Positively stained cells were counted within a tissue area, which was calculated using ImageJ software (National Institutes of Health), and numerous random fields were assessed per tissue section.

### Wet-to-dry weight ratio

The middle lobe of the right lung was harvested at 72 h after intranasal exposure to *S. pneumoniae* or PBS. The freshly isolated middle lobe was weighed to obtain the wet weight, using a precision electronic balance (Sartorius). Then the lung tissues were immediately incubated at 60 °C for 72 h to obtain the dry weight. Finally, the lung wet/dry ratio was calculated.

### Plasma and BALF for luminex assays of cytokines/chemokines

Whole blood was collected 24 h after CLP surgery by cardiac puncture and placed in microtainer tubes containing EDTA (Terumo). Samples were centrifuged for 15 min at 2,000 × g and resulting plasma supernatant was frozen at − 80 °C until use. BALF was collected at 72 h after *S. pneumonia* lung infection. Undiluted samples of plasma and BALF were used for analysis by Luminex MAGPIX assay system per manufacturer’s instructions [20, 29, 30]. The levels of cytokines and chemokines were determined by standard curve analyses.

### Measurement of liver enzyme activity and creatinine in plasma

The activity of alanine transaminase (ALT) and aspartate aminotransferase **(**AST), and the level of creatinine (Cr), in plasma was measured by commercially available colorimetric assay kits (Abcam).

### Statistics

For data with a normal (Gaussian) distribution, a one-way analysis of variance (ANOVA) was performed when more than two groups were analyzed, with a Tukey post hoc test. In data that did not have a normal distribution, a non-parametric analysis was performed using a Kruskal-Wallis test. For comparisons between two groups with a normal distribution, we used Student’s unpaired t test. Data are expressed as the mean ± SEM. Statistical significance was accepted at *P* < 0.05.

## Results

### Characterization of hTSCs in vitro

**Fig. 1:**
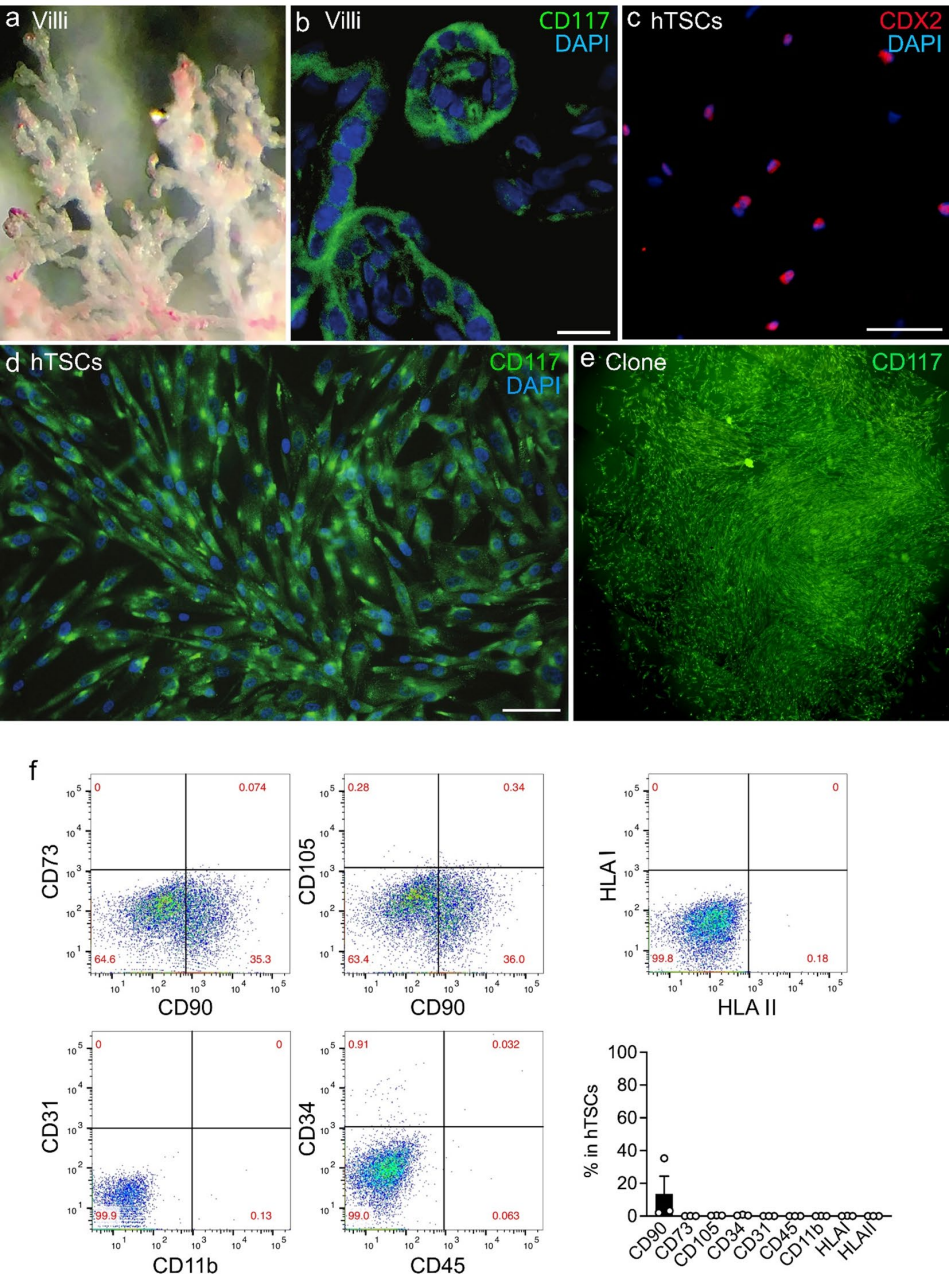
Characterization of CD117^+^ hTSCs in vitro. **(a)** Representative image of human placental chorionic villi. **(b)** Chorionic villi stained positive for CD117 (green), DAPI for nuclear staining (blue). Scale bar presents 10 μm. **(c & d)** hTSCs isolated from the villi express CDX2 (red, **c**), a marker for trophoblast stem cells, and CD117 (green, **d**), DAPI for nuclear staining (blue). Scale bars present 50 μm. **(e)** A hTSC clone stained positive for CD117 in limited dilution culture. Multiple images stitched. **(f)** Representative scatter plots of flow cytometer for CD73 versus CD90 (upper left panel), CD105 versus CD90 (upper middle panel), CD31 versus CD11b (lower left panel) and CD34 versus CD45 (lower right panel). HLA I versus HLA II (upper right panel). Quantitation of the flow cytometric assay in the bar graph show percentage of markers for MSCs (CD90, CD73, CD105) and hematopoietic cells (CD34, CD31, CD45 and CD11b), and HLA I (HLA-A, HLA-B and HLA-C) and HLA II (HLA II: HLA-DR, HLA-DP and HLA-DQ) in the total hTSC population, *n* = 3 for each marker

hTSCs were isolated from the term placental villi after delivery (Fig. 1a). A subpopulation of CD117^+^ cells was present within the cytotrophoblast layer of these placental chorionic villi (Fig. 1b). After expansion of the CD117^+^ cells in vitro, the predominance of the hTSCs expressed caudal type homeobox 2 (CDX2, Fig. 1c), which is crucial for formation and maintenance of the trophoblast [31], and also maintained their expression of CD117 (Fig. 1d). CD117^+^ cells plated at limited dilutions in culture formed clones, which continued to express CD117 (Fig. 1e). These data reveal the stem cell properties of clonogenicity and self-renewal of the hTSCs. We also evaluated additional markers which revealed the absence or very low expression of CD45, CD11b, CD34, and CD31 (Fig. 1f), suggesting that these cells are not of hematopoietic or vascular origin. In addition, the cells expressed very low levels of CD105 and CD73, with < 10% of cells expressing the combination of CD105, CD73, and CD90. Thus the cells do not meet the criteria of MSCs. Finally, the cells did not express HLA I (HLA-A, B, C) and HLA II (HLA-DR, DP, DQ) proteins (Fig. 1f). Moreover, injection of hTSCs i.v. into immunocompetent adult mice resulted in no inflammatory response in the lungs after 1 and 4 days (data not shown), consistent with immune privilege. All of these characteristics are analogous to the previously described mTSCs [21].

### Administration of TSCs after the onset of CLP-induced sepsis promotes improved survival, increased bacterial clearance, and decreased tissue injury

**Fig. 2:**
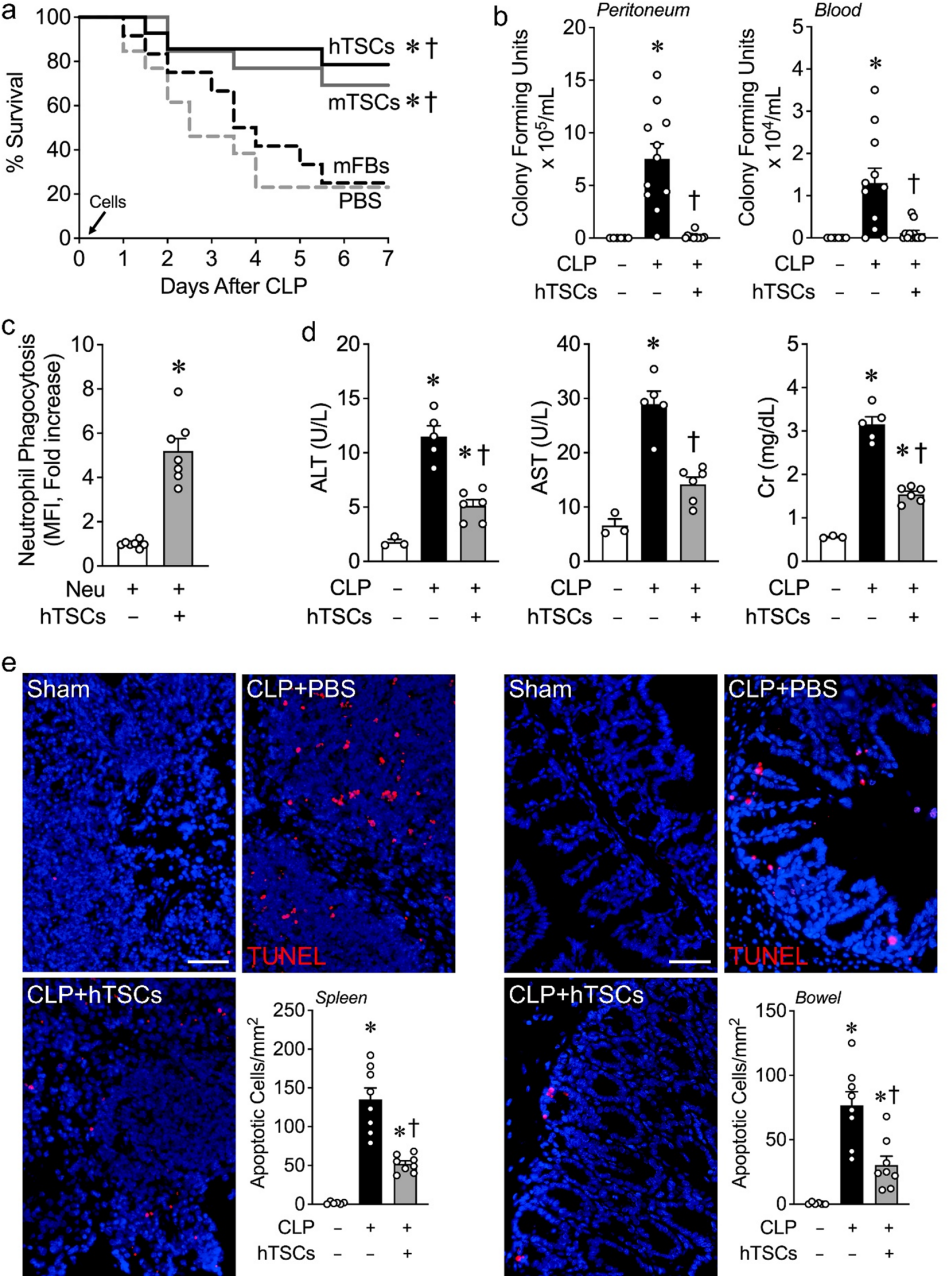
hTSCs promote survival and decrease tissue injury during CLP-induced sepsis. **(a)** Animal survival assay. C57BL/6 mice were subjected to CLP and 6 h later, randomly assigned to receive PBS (*n* = 13), mFBs (*n* = 12), mTSCs (*n* = 13) and hTSCs (*n* = 14). A 5 × 10^5^ cells suspension in 200 µL PBS or PBS was injection via the tail vein. Animal survival was monitored for 7 days, and data are presented as a Kaplan-Meier survival curve. Log-rank test. *P* = 0.0023, overall; *P* ≤ 0.0270 * vs. mFBs, † vs. PBS. No significant difference hTSCs vs. mTSCs, and mFBs vs. PBS. **(b)** Bacterial counts of peritoneal lavage (left panel) and blood (right panel) 24 h after Sham or CLP surgery. Mice were subjected to Sham and received PBS (CLP-hTSCs-) or CLP surgery (CLP+) and received PBS (CLP + hTSCs-) or hTSCs (CLP + hTSCs+), *n* = 6–12 per group. Data are presented as mean ± SEM. Kruskal-Wallis test. *P* ≤ 0.0183, * vs. Sham, † vs. CLP + PBS (hTSCs-). **(c)** Phagocytosis of neutrophils. Activated peritoneal neutrophils were incubated with GFP-labeled *E*.*coli* in the presence of no hTSCs (-) or hTSCs (+) in vitro, *n* = 7 in each group. Data are presented as mean±SEM. Student’s unpaired t test. *P* < 0.0001: * vs. hTSCs-. **(d)** Plasma levels of alanine transaminase (ALT, left panel), aspartate aminotransferase (AST, middle panel), and creatinine (Cr, right panel) were assessed at 24 h after surgery, *n* = 3–6 per group. Data are presented as mean±SEM. One-way ANOVA with Tukey’s post hoc test. *P* ≤ 0.00372, * vs. Sham, † vs. CLP + PBS (hTSCs-). **(e)** Spleen and bowel harvested at 24 h after surgery and immunostaining for TUNEL. Scale bars represent 50 μm. TUNEL quantitation shows in bar graphs, *n* = 6–8 per group. Data are presented as mean±SEM. One-way ANOVA with Tukey’s post hoc test. *P* ≤ 0.0469: * vs. Sham (CLP-), † vs. CLP + PBS (hTSCs-)

To elucidate the effects of TSCs on experimental sepsis, CLP was performed and then 6 h later the mice received either hTSCs, mTSCs, or mFBs at a dose of 5 × 10^5^/200 µL PBS, or vehicle (PBS, 200 µL). Mice receiving vehicle (PBS) or mFBs had survival rates of 23.1% and 25.0% respectively over a 7-day period (Fig. 2a). Administration of hTSCs or mTSCs after the onset of sepsis led to a marked increase in survival to 78.5% or 69.2% respectively, which was significantly improved compared with PBS and mFBs (Fig. 2a). There were no significant differences between the survival of mice receiving hTSCs or mTSCs. Thus, moving forward, we focused on the impact of hTSCs on experimental sepsis.

We next assessed the effect of hTSCs on bacterial clearance in vivo after the onset of CLP-induced sepsis. Mice underwent either Sham (CLP-) or CLP surgery (CLP+). Six hours after surgery, the mice were administered PBS (hTSCs-) or hTSCs (+). After 24 h, bacterial counts were significantly increased from the PF and blood of mice after CLP + PBS, compared with Sham surgery (Fig. 2b). However, mice receiving hTSCs after the onset of CLP-induced sepsis had a marked reduction in bacterial counts compared with mice receiving PBS. We next assessed the effect of neutrophil phagocytosis of bacteria (*E. coli*) in vitro, in the presence or absence of hTSCs. Co-culture of neutrophils with hTSCs produced a significant increase in bacterial phagocytosis compared to neutrophils not exposed to hTSCs (Fig. 2c).

To further understand the injury response, we assessed organ impairment at 24 h after the onset of CLP-induced sepsis. Measurement of the enzymes ALT and AST were done to evaluate liver function, while Cr levels were measured to assess kidney function. AST, ALT, and Cr were all increased in the plasma of septic mice after CLP surgery receiving PBS, compared with mice undergoing Sham surgery (Fig. 2d). These data demonstrate injury to the liver and kidneys. However, in septic mice receiving hTSCs, there was a significant reduction in ALT, AST, and Cr compared with mice after CLP + PBS. Tissue injury in the spleen and bowel were next evaluated by TUNEL staining 24 h after Sham or CLP surgery to assess cell death/apoptosis. Mice receiving hTSCs after the onset of sepsis resulted in a decrease in TUNEL-positive staining in the spleen and bowel (ileum) compared to septic mice receiving PBS (Fig. 2e). Taken together, these data demonstrate that administration of hTSCs after the onset of CLP-induced sepsis results in decreased injury to the liver, kidney, spleen, and bowel compared with mice receiving PBS.

### Inflammatory response in the peritoneal cavity in mice receiving hTSCs after onset of CLP-induced sepsis

**Fig. 3:**
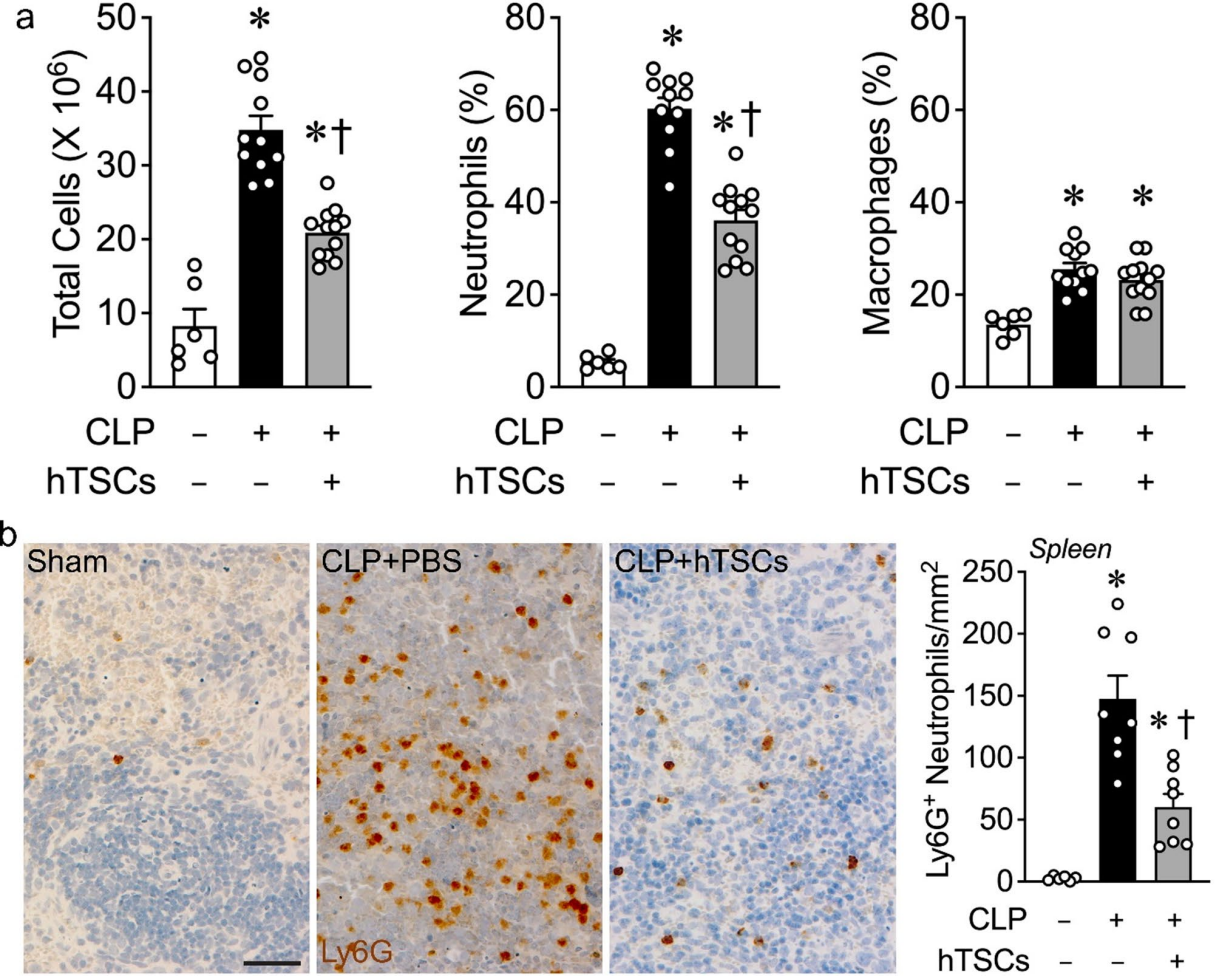
hTSCs attenuate the inflammation response during CLP-induced sepsis. Sham and CLP surgeries were performed on mice, and 6 h later, Sham mice received PBS (CLP-hTSCs-) and CLP mice received PBS (CLP + hTSCs-), or TSCs (CLP + hTSCs+). Peritoneal fluid and spleens were collected at 24 h after surgery. **(a)** Total cell number by counting (left panel), percentage of neutrophils (middle panel) and macrophages (right panel) by flow cytometry assay, in peritoneal fluid, *n* = 6–12 per group. Data are presented as mean±SEM. One-way ANOVA with Tukey’s post hoc test. *P* ≤ 0.0003: * vs. Sham (CLP-), † vs. CLP + PBS (hTSCs-). **(b)** The spleen immunostaining for Ly6G, Scale bar represents 50 μm. The quantitation shows in bar graph, *n* = 6–8 per group. Data are presented as mean±SEM. One-way ANOVA with Tukey’s post hoc test. *P* ≤ 0.0235: * vs. Sham (CLP-), † vs. CLP + PBS (hTSCs-)

We assessed the innate inflammatory cell response in the peritoneum during CLP-induced sepsis, in the presence or absence of hTSC administration. Peritoneal lavages were performed at 24 h after Sham or CLP surgery, and total cell counts as well as percentage of neutrophils and macrophages were performed. There was a dramatic increase in peritoneal total cells counts in mice receiving PBS at 24 h after CLP, compared with Sham surgery (Fig. 3a, left). In mice receiving hTSCs, the total number of cells was significantly decreased compared with CLP mice receiving PBS. Neutrophils were responsible for the majority of this increase in inflammatory cells in the peritoneum, which were decreased in mice injected with hTSCs (Fig. 3a, middle). In contrast, a much smaller fraction of the cells was due to an increase in CD 68-positive monocyte/macrophage lineage cells in the peritoneum of mice after CLP, but there was no difference CD68-positive cell counts in mice receiving PBS or hTSCs at 24 h after CLP (Fig. 3a, right). We next assessed the infiltration of Ly6G-positive neutrophils into the splenic tissue of mice 24 h after Sham or CLP surgery (Fig. 3b). There was a marked increase in neutrophils infiltrating into the splenic tissue 24 h after the onset of CLP-induced surgery, compared with Sham. However, the administration of hTSCs after the onset of sepsis resulted in a marked reduction of neutrophils (Fig. 3b). This decrease in neutrophils after hTSC administration corresponded with improved bacterial clearance and a decrease in tissue apoptosis (Fig. 2), consistent with a resolution of the inflammatory response in mice after receiving hTSCs.

### Systemic inflammatory cytokines and chemokines in mice receiving hTSCs after the onset of CLP-induced sepsis

**Fig. 4:**
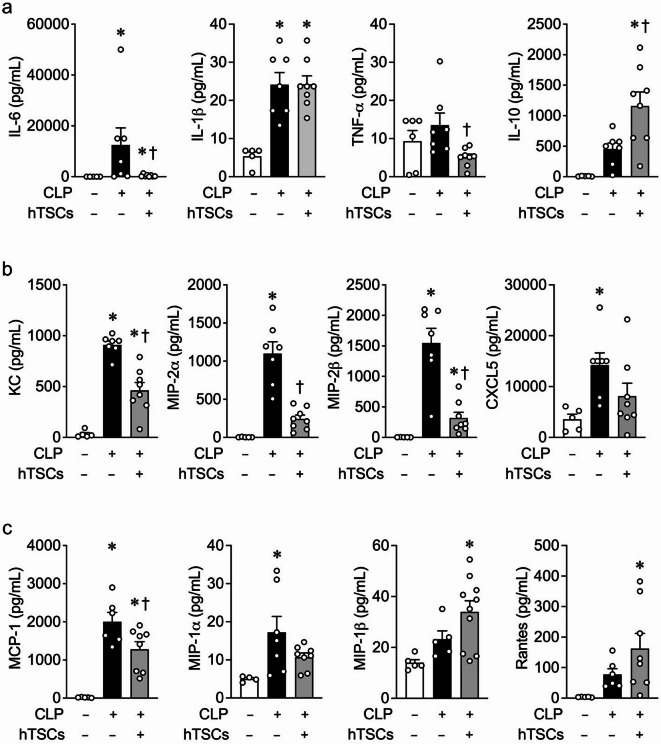
hTSCs regulate systemic inflammatory cytokines and chemokines during CLP-induced sepsis. Luminex assay of plasma from mice 24 h after Sham (CLP-) or CLP surgery (+), received PBS (hTSCs-) or hTSCs (+). Plasma levels of **(a)** pro- and anti-inflammatory cytokines (IL-6, IL-1β, TNF-α and IL-10). **(b)** neutrophil chemokines (KC, MIP-2α, MIP-2β, and CXCL5). **(c)** macrophage chemokines (MCP-1, MIP-1α, MIP-1β, and Rantes). Data are presented as mean±SEM. One-way ANOVA with Tukey’s post hoc test was performed for IL-1β, TNF-α, IL-10, KC, MIP-2α, MIP-1α, MIP-1β, MCP-1, and Rantes. Kruskal-Wallis test was performed for IL-6, MIP-2β, and CXCL5. *n* = 4–8 per group,*P* ≤ 0.0479 for **(a)**; *n* = 5–10 per group, *P* ≤ 0.0003 for **(b)**; *n* = 4–10 per group, *P* ≤ 0.0404 for **(c)**. * vs. Sham (CLP-), † vs. CLP + PBS (hTSCs-)

Cytokines and chemokines are critical mediators of the immune response during sepsis, leading to tissue injury and organ dysfunction [32, 33]. Expression of the pro-inflammatory cytokine interleukin (IL)-6, known to be involved in the pathobiology of sepsis [34–36], is increased in the plasma of mice 24 h after onset of CLP-induced sepsis compared with Sham surgery, and administration of hTSCs to septic mice led to a significant reduction in IL-6 levels (Fig. 4a). There was also a significant decrease in tumor necrosis factor (TNF)-α in septic mice receiving hTSCs compared with PBS, although IL-1β did not decrease. Other inflammatory cytokines such as IL-1α, IL-2, IL-13, IL-23, and interferon (IFN)-γ were not decreased in septic mice receiving hTSCs (Additional file 1). In contrast, the anti-inflammatory cytokine IL-10 was increased in septic mice receiving hTSCs, compared to mice receiving PBS (Fig. 4a). IL-4, which contributes to the polarization of classically activated macrophages (M1) to alternatively activated macrophages (M2) [37], contributing to the resolution of inflammation [38], was also further increased in septic mice receiving hTSCs, compared with septic mice receiving PBS (Additional file 1). In addition, chemokines that contribute to the recruitment of neutrophils, such as keratinocyte-derived chemokine (KC), macrophage inflammatory protein (MIP)-2α, MIP-2β, and C-X-C motif (CXC) 5 were significantly increased in mice with CLP-induced sepsis receiving PBS, compared with Sham surgery, and the administration of hTSCs resulted in a decreased expression in CLP mice, or to a level not different from Sham mice (Fig. 4b). Chemokines important for macrophage recruitment, such as monocyte chemoattractant protein (MCP) -1 and MIP-1α, were also increased in mice after CLP-induced sepsis compared to those that underwent Sham surgery. The administration of hTSCs led to a significant reduction in MCP-1 compared with CLP + PBS; however, MIP-1α remained unchanged. Levels of MIP-1β and Rantes were both increased compared to Sham, but were not different from CLP + PBS (Fig. 4c).

### Administration of hTSCs after the onset of lung infection with *S. pneumoniae* leads to improved bacterial clearance and less injury in the lung

**Fig. 5:**
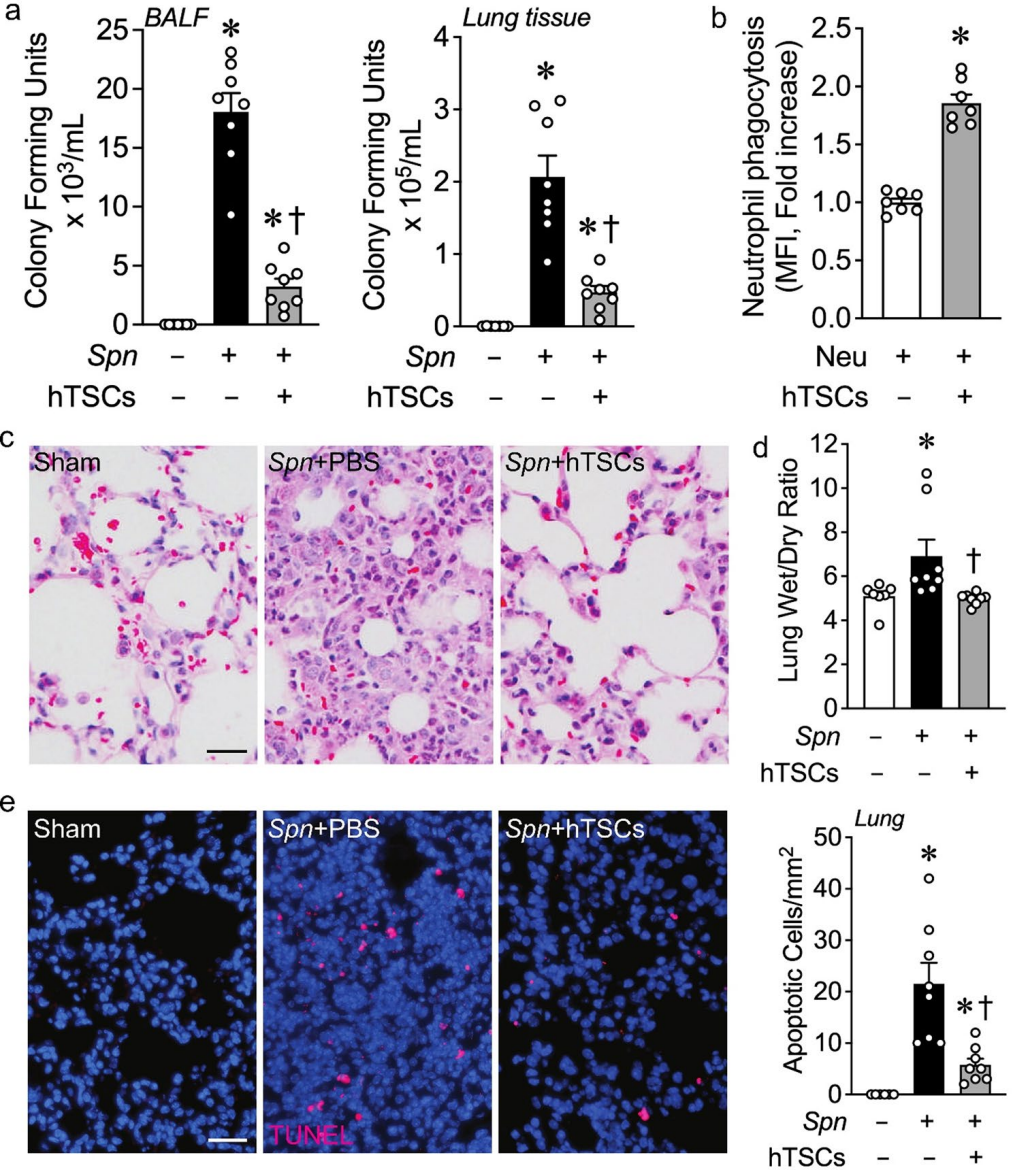
hTSCs promote bacterial clearance and protect tissue from injury during *S. pneumoniae (Spn)*-induced pneumonia. C57BL/6 mice received intranasal inoculation of PBS (*Spn*-, Sham) or *S. pneumoniae* (*Spn*+), and 6 h later, the mice were treated with PBS (hTSCs-) or hTSCs (+) by tail vein injection. BALF and lung tissues were collected at 72 h after the procedure. **(a)** Bacterial counts (CFUs) in BALF (left panel) and lung tissue (right panel), *n* = 7–8 per group. Data are presented as mean±SEM. Kruskal-Wallis test. *P* ≤ 0.0318, * vs. Sham (*Spn*-), † vs. *Spn* + PBS (hTSCs-). **(b)** Phagocytosis of *Spn* by neutrophils. Activated peritoneal neutrophils were incubated with FITC-labeled *Spn* in the presence of no hTSCs (-) or hTSCs (+) in vitro, and flow cytometry was performed. Data are presented as mean±SEM, *n* = 7 per group. Student’s unpaired t test. * *P* < 0.0001 vs. absence of hTSCs (-); **(c)** Representative images of H&E staining of the lungs. Scale bar presents 50 μm. **(d)** Wet to dry weight ratio, an index of pulmonary edema, *n* = 7–8 per group. Data are presented as means±SEM. Kruskal-Wallis test. *P* ≤ 0.0105, * vs. Sham (*Spn*-), † vs. *Spn* + PBS (hTSCs-); **(e)** Representative image of lung staining for TUNEL, Scale bar presents 50 μm. The quantitation shows in bar graph, *n* = 6–8 per group. Data are presented as mean±SEM. Kruskal-Wallis test. *P* ≤ 0.0011, * vs. Sham (*Spn*-), † vs. *Spn* + PBS (hTSCs-)

We next assessed the effect of hTSCs on bacterial clearance from the lungs 72 h after inoculation with *S. pneumoniae*. BALF collected from the left lungs of mice, and tissue harvested from the superior lobes of the right lungs, were evaluated for bacterial counts. There was an increase in bacterial counts from the BALF and lung tissue 72 h after infection with *S. pneumoniae (Spn)* compared with inoculation of PBS (Fig. 5a). Administration of hTSCs 6 h after onset of lung infections resulted in a marked decreased in bacterial counts. Moreover, assessment of *Spn* phagocytosis revealed hTSCs increased the phagocytosis of bacteria by neutrophils, compared with neutrophils not exposed to hTSCs (Fig. 5b).

Assessment of lung morphology was next performed with H&E staining of the lungs. Mice receiving *Spn* + PBS demonstrated interstitial edema and alveolar exudates compared with Sham mice, consistent with pneumonia, and the lung morphology in mice receiving *Spn* + hTSCs showed a reduction in this response, more comparable to Sham (Fig. 5c). Edema was confirmed by lung wet-to-dry weight ratios, with an increased wet/dry ratio in mice inoculated with *Spn*, and administration of hTSCs significantly reduced this ratio to a level analogous to Sham mice (Fig. 5d). Finally, we performed TUNEL staining to assess lung injury. Lungs from mice receiving *Spn* + PBS had increased TUNEL-positive cells compared with Sham lungs, and mice administered *Spn* + hTSCs resulted in a significant decrease in TUNEL-positive cells (Fig. 5e).

### Impact of hTSCs on the inflammatory response in the lung after *S. pneumoniae* infection

**Fig. 6:**
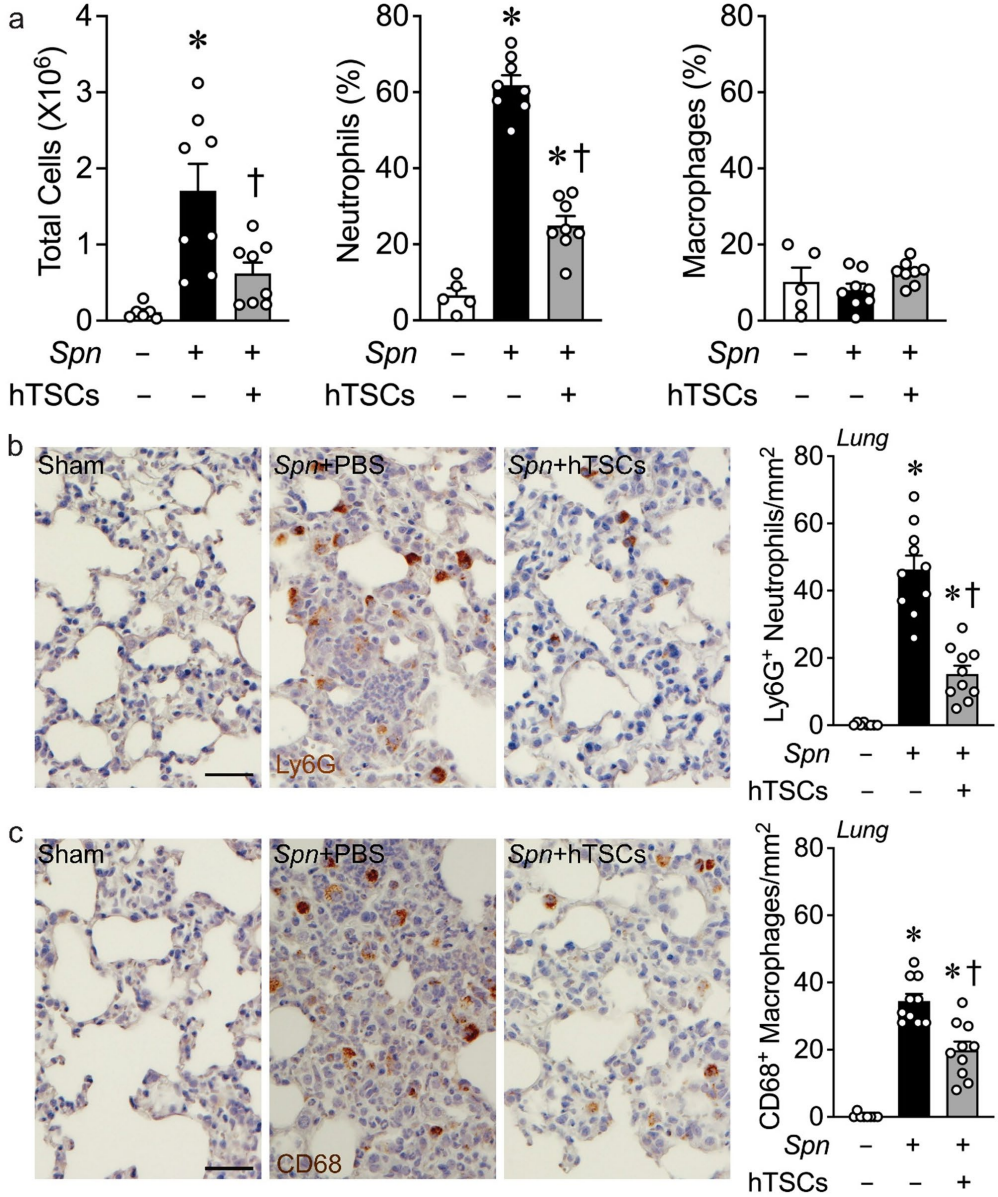
hTSCs reduce the lung inflammatory response during *S. pneumoniae (Spn)*-induced pneumonia. Mice received intranasal inoculation of PBS (*Spn*-, Sham) or *S. pneumoniae* (*Spn*+), and were treated with PBS (hTSCs-) or hTSCs (+) by tail vein injection, 6 h after procedure. BALF and lung tissues were collected at 72 h after lung infection. **(a)** Total cell number by counting (left panel), percentage of neutrophils (middle panel) and macrophages (right panel) by flow cytometry assay in BALF, *n* = 5–8 per group. Data are presented as mean±SEM. One-way ANOVA with Tukey’s post hoc test. *P* ≤ 0.0103: * vs. Sham (*Spn*-), † vs. *Spn* + PBS (hTSCs-); **(b-c)** Representative images of lung immunostaining for Ly6G (neutrophils, **b**) and CD68 (macrophages, **c**). Scale bar represents 50 μm. Quantification is shown in the bar graphs, *n* = 8–10 per group. Data are presented as mean±SEM. One-way ANOVA with Tukey’s post hoc test. *P* ≤ 0.005, * vs. Sham (*Spn*-), † vs. *Spn* + PBS (hTSCs-)

At 72 h after the onset of infection, the overall cell counts in the BALF were markedly increased in the *Spn* + PBS mice compared to Sham, and hTSCs given 6 h after the onset of infection resulted in a significant decrease in total cells (Fig. 6a, left). Assessment of innate inflammatory cells in the BALF showed that the predominant cell type at 72 h remained neutrophils, which were markedly increased in *Spn* + PBS, but were decreased in *Spn* + hTSCs mice compared to mice not receiving hTSCs (Fig. 6a, middle). In contrast, at this time point there was no increase in CD68-positive monocytes/macrophages in the BALF of mice inoculated with *Spn* compared with Sham mice (Fig. 6a, right).

We also assessed innate inflammatory cells that have infiltrated into lung tissue. The tissue from *Spn* + PBS mice had a marked increase in Ly6G-positive neutrophils compared with Sham lungs, and the infiltrating neutrophils were significantly reduced in *Spn* + hTSC lungs (Fig. 6b). Interestingly, CD68-positive macrophages infiltrating into lung tissue were also increased in *Spn* + PBS mice, and administration of hTSCs resulted in a significant decrease in the CD68-positive cells (Fig. 6c).

### Inflammatory cytokines and chemokines in BALF of mice with lung infection due to *S. pneumoniae*

**Fig. 7:**
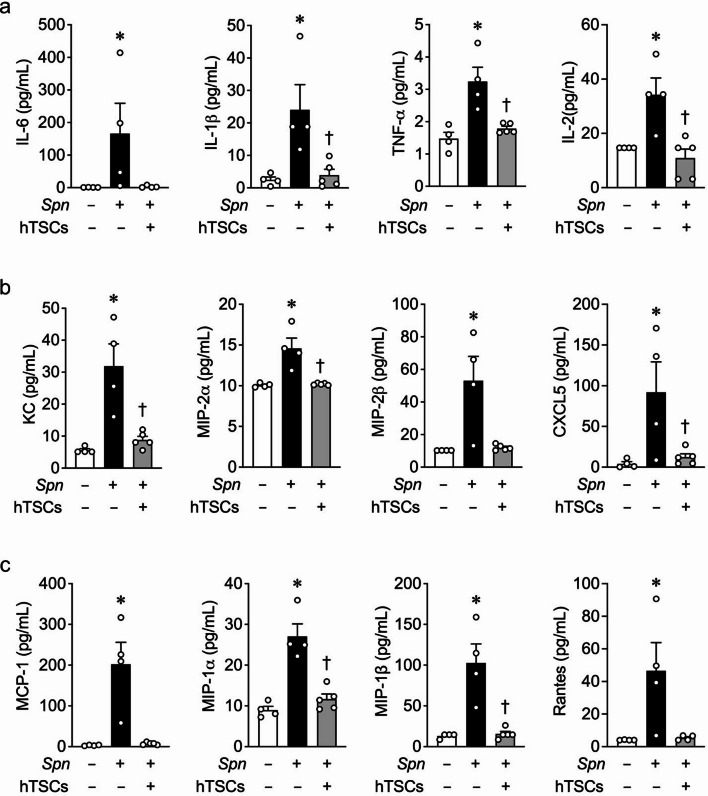
hTSCs regulate inflammatory cytokines and chemokines in BALF during *S*. *pneumoniae* (*Spn*)-induced pneumonia. Luminex assay of BALF from mice 72 h after inoculation with PBS (Sham) or *Spn*, received PBS (hTSCs-) or hTSCs (+). **(a)** pro-inflammatory cytokines (IL-6, IL-1β, TNF-α and IL-2). **(b)** neutrophil chemokines (KC, MIP-2α, MIP-2β and CXCL5). **(c)** macrophages chemokines (MCP-1, MIP-1α, MIP-1β and Rantes). Data are presented as mean±SEM. One-way ANOVA with Tukey’s post hoc test was performed for TNF-α, IL-2, KC, CXCL5, and MIP-1α. Kruskal-Wallis test was performed for IL-6, IL-1β, MIP-2α, MIP-2β, MCP-1, MIP-1β, and Rantes. *n* = 4–5 per group, *P* ≤ 0.0464 for **(a)**; *n* = 4–5 per group, *P* ≤ 0.0394 for **(b)**; *n* = 4 per group, *P* ≤ 0.0417 for **(c)**. * vs. Sham (*Spn*-), † vs. *Spn* + PBS (hTSCs-)

The pro-inflammatory cytokines IL-6, IL-1β, TNF-α, and IL-2 were all significantly increased in BALF from *Spn* + PBS mice, while in mice receiving *Spn* + hTSCs these cytokines were decreased in the BALF compared with *Spn* + PBS mice (Fig. 7a). IL-1α and IL-23 were also increased in *Spn* + PBS mice, but not different from Sham in *Spn* + hTSCs mice (Additional file 2). In contrast to mice with CLP receiving PBS, in mice administered *Spn* + PBS the level of IL10 was below the level of assay detectability. The neutrophil chemokines KC, MIP-2α, MIP-2β, and CXCL5 were all significantly increased in mice receiving *Spn* + PBS, and these chemokine levels were decreased to levels not different from Sham in *Spn* + hTSCs mice (Fig. 7b). Moreover, macrophage chemokines MCP-1, MIP-1α, MIP-1β, and Rantes were significantly increased in BALF from *Spn* + PBS, while in BALF of *Spn* + hTSCs mice these chemokines were reduced to a level not different from Sham mice (Fig. 7c).

**Fig. 8:**
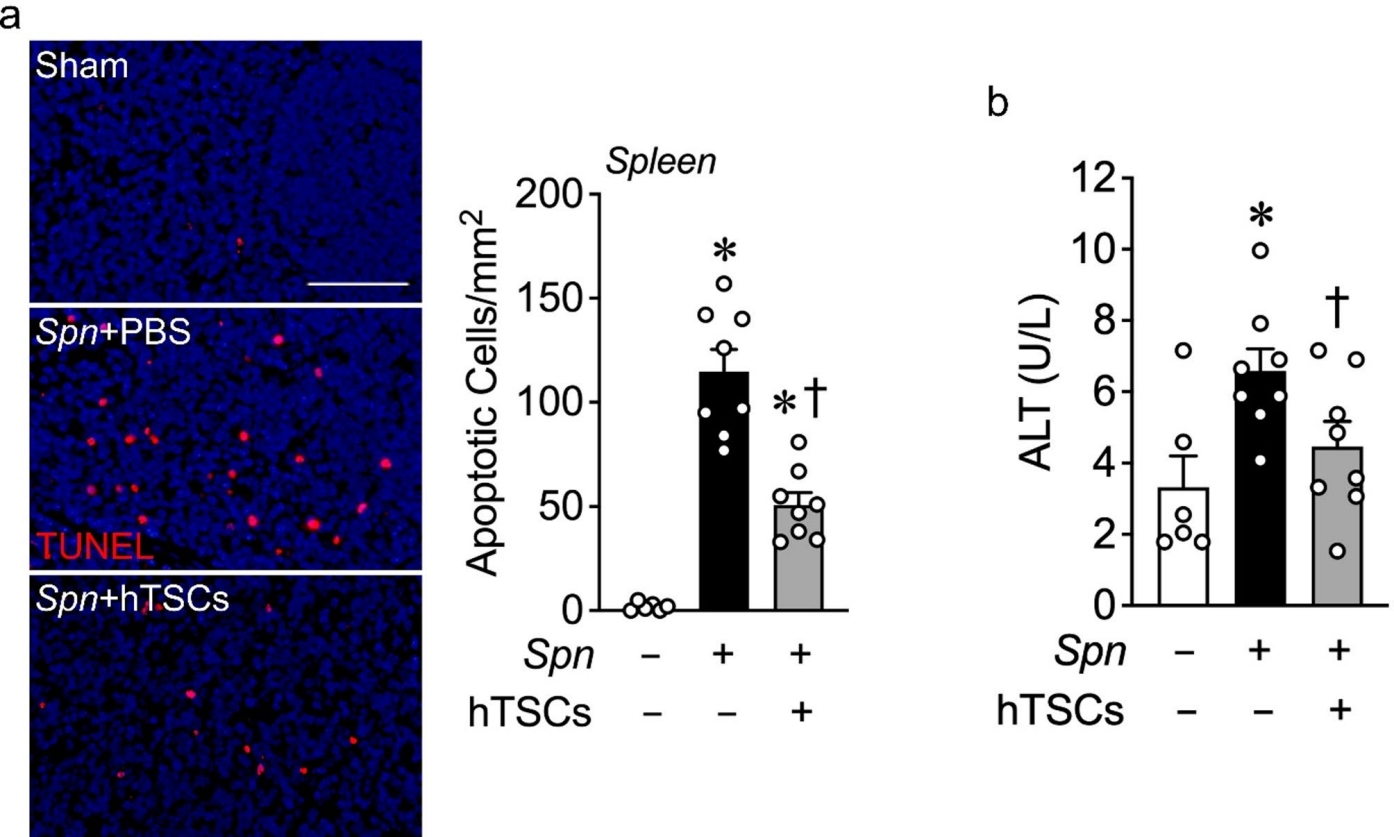
hTSCs decrease systemic organ injury during *S. pneumoniae* (*Spn*) lung infection. Lungs of mice were inoculated with PBS (Sham, *Spn*-) or *Spn* (+) and following by treatment of PBS (hTSCs-) or hTSCs (+) 6 h later. Plasma and spleen were harvested at 72 h after *Spn* lung infection. **(a)** Representative image of spleens staining for TUNEL. Scale bar represents 100 μm. Quantitative data is shown in the bar graph, *n* = 6–8 per group. Data are presented as mean±SEM. One-way ANOVA with Tukey’s post hoc test. *P* ≤ 0.001: * vs. Sham (*Spn*-), † vs. *Spn* + PBS (hTSCs-). **(b)** Alanine aminotransferase (ALT) activity in plasma was measured by a commercial assay kit (Abcam), *n* = 6–8 per group. Data are presented as mean ± SEM. One-way ANOVA with Tukey’s post hoc test. *P* ≤ 0.0455: * vs. Sham (Spn-), † vs. Spn + PBS (hTSCs-)

Finally, we verified whether the mice intranasally inoculated with *S. pneumoniae* solely resulted in a pneumonia, or also had a systemic process. Assessment of TUNEL staining in the spleen noted an increase in TUNEL-positive cells in *Spn* + PBS mice compared with Sham, which was decreased in *Spn* + hTSCs mice (Fig. 8a). Moreover, evaluation of ALT in mice receiving *Spn* + PBS demonstrated an increase in ALT, while mice administered *Spn* + hTSCs had a decrease in ALT levels analogous to Sham mice (Fig. 8b). Taken together these data demonstrate a systemic response to lung infection by *S. pneumoniae*, resulting in injury to both the liver and spleen. These data reveal that the *S. pneumoniae* infection not only led to a pneumonia, but also resulted in pneumosepsis, which was improved with less injury after the administration of hTSCs.

## Discussion

Due to the therapeutic challenges related to sepsis and its associated organ injuries, there is interest in exploring new treatment strategies for this heterogeneous disease process. Thus, our emphasis on exploring a new cell-based approach, in which viable therapeutic cells sense the underlying septic environment, and respond accordingly with varied actions. The placenta is an organ that is discarded after a normal birth, and also is a reservoir for a variety of stem and progenitor cells [39] that may be applied for use therapeutically. We recently identified CD117^+^ TSCs derived from the fetal portion of near-term mouse placentas [21]. These cells, when administered to mice after bleomycin-induced acute lung injury, resulted in a decrease in inflammation and lung injury [22]. Properties of these cells, including their immune privilege and immunomodulatory capability, led us to explore the presence of an analogous human cell.

Cells have been derived from the trophectoderm of blastocysts and first-trimester placentas [40, 41], which meet the criteria for human trophoblast stem cells. However, isolating TSCs from human mature placentas has been a challenge [42]. Recently, the isolation of multipotent cells from the chorionic regions of human term placentas was reported, targeting the non-cell surface protein CDX2 expressing cells [43]. We focused on the chorionic villi from the fetal portion of the placenta (≈ 95% of cells of fetal origin [44]), but instead used CD117, a cell surface receptor tyrosine kinase, as a means to isolate the putative stem cells. A subpopulation of CD117^+^ cells was identified in the cytotrophoblast layer of chorionic villi, and these cells also expressed CDX2 and maintained the stem cell properties of clonogenicity and self-renewal. Moreover, the cells were not of hematopoietic or vascular origin, and did not meet the criteria of MSCs.

Much of the prior data regarding hTSCs and their impact on infections has focused on placental pathobiology. For instance, hTSCs and trophoblast organoids provided an approach to model viral infections during early development leading to placental injury [45–47]. However, to our knowledge, the present studies demonstrate the first use of hTSCs as a cellular therapy in preclinical models of bacterial infection and sepsis. We selected both a polymicrobial model of CLP [23] and a single organism pneumonia model due to *S. pneumoniae* [27], both of which result in sepsis and organ injury, to explore the impact of CD117^+^ hTSCs. The CLP model mimics clinical peritonitis from appendicitis or diverticulitis resulting in bowel perforation or bacteria translocation into the peritoneal cavity. In regard to *S. pneumoniae*, it is the most commonly identified bacterial cause of community-acquired pneumonia [48]. Mortality associated with pneumococcal pneumonia in hospitalized patients is high, ranging from 12 to 30%. Moreover, bacteremia complicates pneumococcal pneumonia in approximately 25% of cases [49]. Thus, we also investigated a *S. pneumoniae* model of pneumonia and subsequent sepsis [24], often complicated by lung injury and ARDS.

CLP, depending on the length of the cecum ligated and the number and size of holes, is a very aggressive model of polymicrobial sepsis, with onset of death as early as the first 24 h. CD117^+^ hTSCs and mTSCs were both very effective in preventing death when administered 6 h after the onset of sepsis compared with the administration of mFBs (control cell) or PBS (same vehicle volume used for cell injections). To further understand the impact of TSCs on CLP-induced sepsis, we focused on hTSCs, to advance our understanding of a human cell-based approach using mouse models. This was feasible due to the immune privileged nature of hTSCs.

Sepsis has been defined as organ dysfunction caused by a dysregulated host response to severe infection [50]. The initial stage of sepsis leads to a hyperinflammatory response through the recognition of pathogens and damaged tissue, resulting in systemic activation of the innate immune system with release of pro-inflammatory cytokines [51]. In the CLP model, an exaggerated neutrophil response in the peritoneum occurs within 24 h of surgery, and the hTSCs are able to suppress this response, although not entirely back to the level of baseline. In addition, there is a marked decrease in tissue neutrophils as seen in the spleen. Assessment of circulating pro-inflammatory cytokines and chemokines, especially those contributing to neutrophil recruitment, are decreased in mice receiving hTSCs compared with mice receiving vehicle. A compensatory increase in IL-10, an anti-inflammatory cytokine, is known to correlate with severity of the inflammatory response and associated organ failure in severe sepsis [52, 53]. Thus, the trend for an increase in IL-10 in the CLP mice followed this concept, and the IL-10 levels were further increased in mice receiving hTSCs after the onset of CLP-induced sepsis. At the time of decreased pro-inflammatory cytokines and chemokines, and increased anti-inflammatory mediators such as IL-10, hTSCs had already led to a marked bacterial clearance and decreased injury in the liver, kidney, spleen, and bowel. Moreover, over a 7 day period, TSCs (both human and murine) resulted in a dramatic increase in survival. Thus, the ability of hTSCs to respond to the septic milieu led to a beneficial immunomodulation response in CLP-induced sepsis after a single dose, and does not suggest prolonged immunosuppression, which may be detrimental. Interestingly, hTSCs also promote an increase in IL-4, which not only contributes to inhibition of an acute inflammatory response and promotes resolution of inflammation, but can also induce long-lasting trained immunity [54].

To appreciate the impact of hTSCs more fully on lung injury due to infection, we turned to a *S. pneumoniae* model of pneumonia [27]. This provided not only a second model, but also a different primary pathogen than occurs with CLP, as *S. pneumoniae* is not an organism typically arising from the bowel flora [55]. CD117^+^ hTSCs were able to significantly improve bacterial clearance in the lung at 72 h after infection, in part by increasing neutrophil phagocytosis of bacteria. This was associated with a decrease in lung edema and parenchymal cell death. Moreover, the marked increase in cells in the BALF after *S. pneumoniae* infection was predominantly neutrophils, and this response was suppressed by hTSCs at a time when bacterial counts were not significantly different than Sham mice. Moreover, infiltrating neutrophils and macrophages in the lung parenchyma were reduced in mice administered hTSCs compared with mice receiving vehicle. This corresponded with a decrease in pro-inflammatory cytokines and chemokines in BALF. Thus, in the pneumonia model, the hTSCs were similarly able to promote the clearance of bacteria, and with their immunomodulatory properties, initiate the resolution of inflammation. While we previously demonstrated that intratracheal mTSCs were able to attenuate acute lung injury due to bleomycin [22], we now reveal that hTSCs given i.v. decreased lung injury related to *S. pneumoniae* infection and the associated inflammatory response. Finally, this model of pneumonia due to *S. pneumoniae* also led to a systemic sepsis response, with an increase in liver and spleen injury, that was averted by the administration of hTSC.

The present study provides data to support the therapeutic impact of hTSCs in experimental models of sepsis. We are presently limited to the use of mouse models of disease. In addition, hTSCs were not used in conjunction with antibiotics and hemodynamic resuscitation, supportive care for human patients, and will need to be tested further in the future. While we believe that hTSCs have the potential for future therapeutic application, they are not yet suitable for clinical translation at this early stage of investigation.

## Conclusion

Collectively, we have demonstrated for the first time that CD117^+^ hTSCs, isolated from human term placentas, have a beneficial therapeutic response in two preclinical models of bacterial infection and sepsis. In each model, hTSCs promoted bacterial clearance, and their immunomodulatory properties supported resolution of the inflammatory response, resulting in protection from organ injury. This is a critical advancement, as CD117^+^ hTSCs were harvested from term placentas, which provide a readily available source of stem cells with no ethical concerns. Moreover, their immune privileged nature, allows for the use of allogeneic cells that can respond to a heterogenous disease process.

## Electronic supplementary material

Below is the link to the electronic supplementary material.

Supplementary Material 1

Supplementary Material 2

## Data Availability

All data generated or analyzed during the current study are included in this published article, and its supplementary information files.

## Abbreviations

ALT: alanine transaminase
AST: aspartate aminotransferase
ARDS: acute respiratory distress syndrome
BALF: bronchoalveolar lavage fluid
CDX2: caudal type homeobox 2
CFUs: colony-forming units
CLP: cecal ligation and puncture
Cr: creatinine
CXC: C-X-C motif ligand
DAPI: 4’,6-diamidino-2-phenylindole
EDTA: ethylenediaminetetraacetic acid
E. coli: Escherichia coli
FBs: fibroblasts
FITC: fluorescein isothiocyanate
G-CSF: Granulocyte-Colony Stimulating Factor
HBSS: Hanks’ Balanced Salt Solution
HEPES: 4-(2-hydroxyethyl)-1-piperazineethanesulfonic acid
H: human
HLA: human leukocyte antigen
IL: interleukin
M: mouse or murine
KC: keratinocyte-derived chemokine
M1: classically activated macrophages
M2: alternatively activated macrophages
MCP: monocyte chemoattractant protein
MIP: macrophage inflammatory protein
MSCs: mesenchymal stem/stromal cells
NaHCO3: sodium bicarbonate
PF: peritoneal fluid
S. pneumoniae or Spn: Streptococcus pneumoniae
TSCs: trophoblast stem cells
TNF: tumor necrosis factor
TUNEL: terminal deoxynucleotidyl transferase-mediated dUTP nick end-labeling
