# Supplementary material for: Therapeutic impact of human trophoblast stem cells in peritoneal and pneumonia-induced sepsis in mice
Source: Stem Cell Res Ther. 2025 Jul 21;16:394. doi: 10.1186/s13287-025-04479-z (PMC12282005; doi:10.1186/s13287-025-04479-z)
Supplement: Supplementary file 1 — Supplementary Material 1 [file 13287_2025_4479_MOESM1_ESM.pdf]

Additional file 1

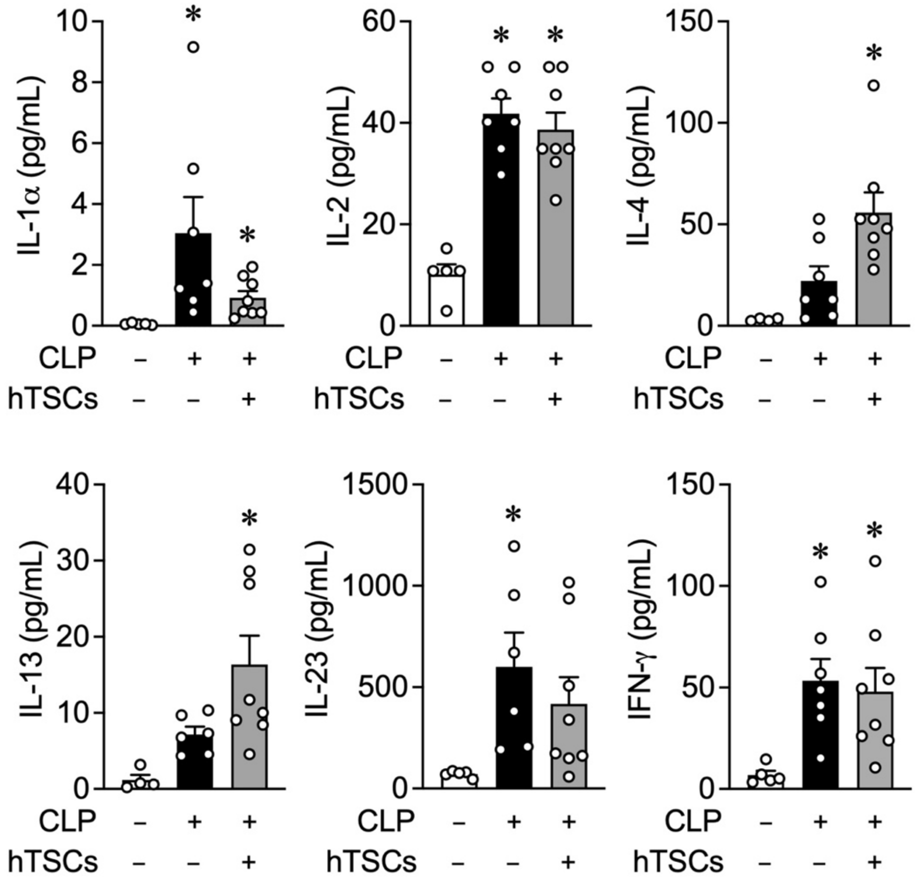

Additional file 1. hTSCs regulate systemic cytokines during CLP-induced sepsis. Luminex assay of plasma from mice 24 hours after Sham (CLP-) or CLP surgery (+), received PBS (hTSCs-) or hTSCs (+). Plasma levels of cytokines that regulate the inflammatory response (IL-1α, IL-2, IL-4, IL-13, IL-23, IFN-γ) were assessed, n=4-8 per group. Data are presented as mean±SEM. One-way ANOVA with Tukey's post hoc test was performed for IL-2, IL-13, IL-23, and IFN-γ. Kruskal-Wallis test was performed for IL-1α and IL-4. $P \leq 0.0479$, * vs Sham (CLP-), † vs. CLP+PBS (hTSC-).
