# Supplementary material for: Therapeutic impact of human trophoblast stem cells in peritoneal and pneumonia-induced sepsis in mice
Source: Stem Cell Res Ther. 2025 Jul 21;16:394. doi: 10.1186/s13287-025-04479-z (PMC12282005; doi:10.1186/s13287-025-04479-z)
Supplement: Supplementary file 2 — Supplementary Material 2 [file 13287_2025_4479_MOESM2_ESM.pdf]

Additional file 2

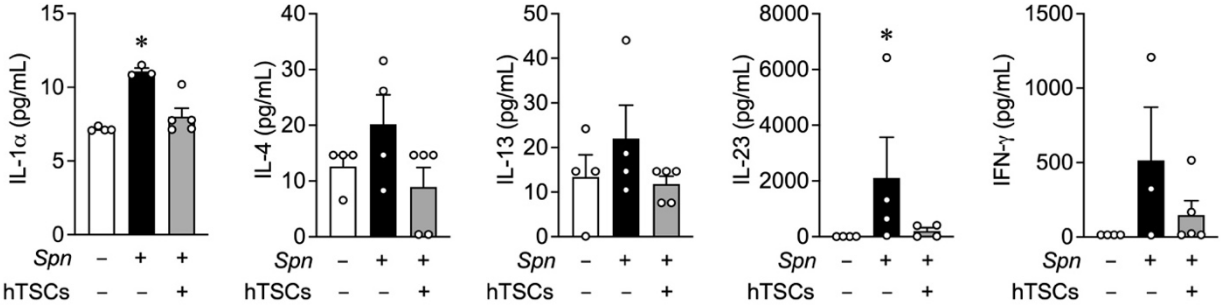

Additional file 2. hTSCs regulate cytokines in BALF during *S. pneumoniae* (*Spn*)-induced pneumonia. Luminex assay of BALF from mice 72 hours after inoculation with PBS (Sham) or *Spn*, received PBS (hTSCs-) or hTSCs (+). BALF levels of cytokines that regulate the inflammatory response (IL-1α, IL-4, IL-13, IL-23, IFN-γ) were assessed, n=3-5 per group. Data are presented as mean±SEM. One-way ANOVA with Tukey's post hoc test was performed for IL-4, IL-13, and IFN-γ. Kruskal-Wallis was performed for IL-1α and IL-23. $P \leq 0.011$, * vs Sham (*Spn*-), † vs *Spn*+PBS (hTSC-).
